# Chemosensory Device Assisted-Estimation of the Quality of Edible Oils with Repetitive Frying

**DOI:** 10.3390/foods10050972

**Published:** 2021-04-29

**Authors:** Jookyeong Lee, Changguk Boo, Seong-jun Hong, Eui-Cheol Shin

**Affiliations:** 1 CASS Food Research Centre, School of Exercise and Nutrition Sciences, Faculty of Health, Deakin University, 221 Burwood Highway, Burwood, VIC 3125, Australia; leejook@deakin.edu.au; 2 Department of Food Science/Institute for Food Sensory & Cognitive Science, Gyeongsang National University, Jinju, Gyeongnam 52725, Korea; dbs7987@naver.com (C.B.); 01028287383a@gmail.com (S.-j.H.)

**Keywords:** frying oil, lipid oxidation, chemical property, sensory quality, electronic tongue, electronic nose

## Abstract

This study investigated chemosensory degradations of soybean and canola oils with repeated frying in order to estimate the quality of the oils. Methods: Chemical parameters including oxygen induction time, acid value, *p*-anisidine value, malondialdehyde, and total polar compounds were measured. Electronic nose and electronic tongue analyses were performed to assess sensory properties. Multivariate analyses were employed to investigate relationships among tastes and volatile compounds using principal component analysis (PCA) and Pearson’s correlation analysis. Results: All chemical parameters increased with repeated frying in both oils. Electronic nose analysis found ethyl butyrate, 2-heptenal, and 2,4-pentanedione as major volatiles for soybean oil and ethyl butyrate and linalool for canola oil. As the numbers of frying increased, all volatiles showed an increased concentration in various extents. In multivariate analyses, ethyl butyrate revealed strong positive correlations with sourness, umami, and sweetness, and umami showed strong positive correlations with sourness and saltiness (*p* < 0.05). PCA confirmed that in PC1 with 49% variance, sourness, saltiness, and umami were at similar rates while acetyl pyrazine, 2,4-pentadieone, and 1-octanol were found at similar rates. Canola oil was chemically more stable and less susceptible to deterioration in all chemical parameters compared to soybean oil, resulting in a relatively better quality oil when repeatedly fried. Conclusion: The results suggested that minimum repeated frying (5 times) degrades chemosensory characteristics of both oils, thereby compromising their quality. The findings of this study will be utilized as a foundation for quality control of fried foods in food industry, fried food development, and fast-food industry.

## 1. Introduction

Frying holds a long history as a classical and common food process, and fried foods have gained popularity due to preferred flavors and textures. Frying is a relatively easy cooking method widely employed in home cooking environments, restaurants, and fast-food industry [1,2]. Fundamentally, heat transfer of frying oil occurs through convection, conduction, or radiation at relatively high temperatures (150–200 °C). Carbohydrates and proteins in foods mainly undergo various physical and chemical degradation during frying at such high temperatures and generate unique sensory characteristics such as palatable mouthfeel, crispy texture, and golden brownish appearance [3]. Of numerous fried foods, deep fried potatoes and chicken are the most prevalently consumed items throughout the world [4]. Both animal- and plant-based oils are used for frying but an elevated interest in health complications leads to an increased utilization of plant-based oils such as olive, sunflower, canola, and soybean oils [4,5].

Among the plant-based oils, canola and soybean oils are commonly used edible oils along with olive and sunflower oils. Canola oil is composed of high monounsaturated fatty acids with 59–61% oleic acid (C18:1). It also contains polyunsaturated fatty acids with 18–21% linoleic acid (C18:2), known as omega-6 fatty acids, as well as 8–11% of α-Linolenic acid (C18:3), known as omega-3 fatty acids [4,5,6,7]. Compared to canola oil, soybean oil possesses a different fatty acid profile with high amounts of linoleic acid (53–56%), followed by oleic acid (17–20%) and linolenic acid (12–15%). Both also contain saturated fatty acids with 3–5% and 9–12% of palmitic acid (C16:0) in canola and soybean oils, respectively [4,5,6,7]. High unsaturated fatty acids and relatively low saturated fatty acid contents in both oils are associated with ranges of health benefits: decrease of LDL cholesterols, increase of HDL cholesterols, increase of insulin resistance, and prevention of cardiovascular diseases, heart disease, and cancer [8]. However, the differences in fatty acid compositions between the two presumably result in varying patterns of heat-induced lipid oxidation and sensory quality deterioration. In general, heat treatment opens up the double bonds of unsaturated fatty acids and therefore, the higher the number of double bonds in the fatty acid chain, the greater the chance of being oxidized during frying. That is, polyunsaturated fatty acids undergo lipid oxidation more easily than monounsaturated fatty acids during frying and thereby soybean oil with a relatively higher proportion of polyunsaturated fatty acids is anticipated to be more prone to lipid oxidation than canola oil [4,5,6,7].

The quality of frying oils substantially influences the safety and quality of fried foods. Frying oils chemically transform during frying processes, which may compromise the quality of oils [1]. In practice, physical changes such as dark color, intense smoke, rancid odor, and soapy appearance of frying oil may be the main indicators that it is the appropriate time to discard the frying oil. Not only physical characteristics but also chemical measures have been used to determine the quality of frying oil [5]. Oxidative and hydrolytic changes of deteriorated oils can be monitored by oxidation measures such as oxidation induction period, acid value, peroxide value, *p*-anisidine value, and total polar compounds [9]. These are conventional measures indirectly estimating oxidative stability and lipid oxidation products, and many studies applied these parameters in monitoring quality of edible oils; total polar compounds and *p*-anisidine value were assessed in frying oils actually used in local restaurants to address their food quality and safety issues [2]; four types of edible oils were subjected to the measurement of acid value and peroxide value in pork cutlets and French fries with repetitive frying [10]; acid value, peroxide value, and oxidation induction time were investigated and compared among plant-based edible oils and forest sourced oils [11].

The changes of oil properties during frying are indicative of alterations of sensory quality [12]. Xu et al. demonstrated that chemical parameters measured by total polar compounds and free fatty acid contents were negatively related to overall sensory acceptability and crispiness and positively related to the greasiness of potato chips when deep fried using canola oil [13]. Another study also showed evidence that increased *p*-anisidine value, total polar compounds, and free fatty acid contents were associated with increased overall rejection, off-flavor/taste, rancid and burnt flavor, and bitterness of potatoes that were fried with rapeseed oil [14]. Similar patterns of sensory deterioration were observed in a study by Santos et al., showing that prolonged frying of potatoes resulted in increased aftertaste and decreased acceptance when deep fried with canola oil, peanut oil, and extra-virgin olive oil [15]. As such, the chemical parameters are increased during frying or repetitive frying and the increased parameters indicate deterioration of overall sensory quality.

In addition to the chemical measures, electronic noses and electronic tongues have been widely used in assessing the safety and sensory quality of foods. Electronic noses and electronic tongues are equipped with electrochemical sensors that imitate human olfactory and gustatory functions, respectively, and provide objective responses to the corresponding stimulus exposures [16,17]. They are also useful in overcoming some of the challenges around sensory testing with human participants such as resource constraints, participant interactions, and data reproducibility [18]. Furthermore, both instruments involve minimum sample preparations and produce immediate outcomes, thereby gaining popularity in the food industry as well as other research areas. Researchers employed an electronic nose and successfully monitored the oxidative stability of rapeseed oil, sunflower oil, and olive oil for quality control of the oils during frying [19,20,21]. Studies also reported that electronic nose analysis is a comparable method in place of conventional chemical measures of oils as mentioned above. In addition to electronic noses, recent studies applied electronic tongues to flavor and aroma profiling of edible oils [19,20,21], coffee [22], wine [23], and meats [24] in purpose of quality monitoring. Studies also found that a combination of electronic nose and electronic tongue is effective in examining chemical deteriorations of edible oils during the frying process [17].

Although many studies have investigated chemical measures of plant-based oils upon frying as a quality indicator, relatively fewer studies have discovered the impacts of repetitive frying on chemical features of plant based-cooking oils as well as sensory characteristics using the electronic nose and electronic tongue, particularly in canola and soybean oils, two of the most commonly used cooking oils in Asian culture. Therefore, the aim of this study was (1) to investigate deteriorations in chemical properties of soybean and canola oils with repetitive frying at 180 °C, (2) to evaluate the changes in sensory characteristics using an electronic nose and electronic tongue, and (3) to examine the interrelationships between the chemical and sensory changes.

## 2. Materials and Methods

### 2.1. Material

Soybean oil, canola oil, and frozen potatoes for French fries (1 cm × 1.5 cm × 10 cm) were purchased from local grocery store (Jinju, Republic of Korea). Each oil (1 L) was placed in a fryer (KFR1201, Kaiser Co., Seoul, Korea) and frozen potatoes (100 g) were fried at 180 °C for 10 min each frying while keeping the cover open during the frying process. The frying was repeated up to 20 times and therefore, the potatoes were fried for 200 min maximum [12]. The potatoes were removed after frying and the frying oil (100 mL) was taken after 5, 10, 15, and 20 times of frying for further analyses. An average 100 mL of fresh oil was added to the fryer after each frying in order to maintain the total volume of 1 L.

### 2.2. Chemical Measures

A Rancimat (892 Professional Rancimat, Metrohm AG, Herisau, Switzerland) instrument was used to assess oxidation induction time of frying oils. A sample of 3 mL was placed in a rancimat tube and air-flushed at 20 L/hr. The system was run at 120 °C and oxygen induction time was determined based on the conductivity of sensors measuring oxidation products generated by oxidation [11].

The acid value of frying oils was determined by the AOCS method. A sample of 1 mL was dissolved in 30 mL of solution mixed with ethanol to ether in 1 to 1(*v*/*v*). Next, 100 μL of 1% phenolphthalein solution was added to the solution as an indicator. Subsequently, the mixture was titrated by 0.1 N potassium hydroxide (KOH)/ethanol solution until the mixture turned to pinkish red [25].

*p*-Anisidine value was determined by dissolving 100 μL of sample in 25 mL of isooctane, followed by measuring absorbance of the mixture at 350 nm. Subsequently, 2.5 mL of the solution was mixed with 0.5 mL of 0.25%(*w*/*v*) *p*-anisidine/acetic acid and reacted for 15 min. Then, absorbance was measured again at 350 nm. Difference in the absorbance before and after the reaction was determined as *p*-anisidine values [26].

Malonealdehyde was determined by the spectroscopic method and HPLC (Agilent 1100, Agilent Co., Santa Clara, CA, USA) analysis. A sample of 10 μL was diluted with 0.1 N HCl and hydrolyzed at 100 °C for 5 min. Stock solution was prepared at each concentration to draw a standard curve and the absorbance was measured at 254 nm. Further, HPLC analysis was conducted using μ-Bondapack C_18_ column. Malonaldehyde content was determined using 250 nm UV wavelengths at 30 °C with mobile phase of acetonitrile and 10% acetic acid at a ratio of 3:1 [27].

Total polar compounds were measured using a cooking oil tester probe (Testo Korea Ltd., Seoul, Korea). The tester probe was placed on the 100 mL of sample at 40–50 °C and total polar compounds were determined by the probe sensors [11].

### 2.3. Sensory Measures

Electronic nose analysis was performed to determine the sensory features of frying oils. Electronic noses utilize multi-sensor arrays to fingerprint the patterns of signals received from sensors that are specific to olfactory compounds [28,29,30]. Samples were prepared based on the instructions provided by the manufacturer (HERACLES-NEO, Alpha MOS, Toulouse, France). First, a 1 mL sample was placed in a headspace vial (22.5 × 75 mm, PTFE/silicon septum, aluminum cap). While stirring at 40 °C and 500 rpm for 5 min, 1000 μL volatile compounds were collected using an automatic sampler and injected into an electronic nose (HERACLES-NEO, Alpha MOS, Toulouse, France) mounted with DB-5 column (30 m × 0.25 mm i.d. × 0.25 μm film thickness) and flame ionization detectors (FID). Hydrogen gas was set to 1 mL/min. The oven temperature was set as follows: held at 40 °C for 5 s, increased to 270 °C at 4 °C/s, and maintained for 30 s. Compounds corresponding to the separated peaks were identified using Kovat’s index-library-based AroChembase (Alpha MOS) [31].

Electronic tongue analysis was conducted to evaluate the five basic tastes in frying oils using an electronic tongue module. The electronic tongue replaces sensory receptors (ion channels) in the human tongue. Sensors mounted in the electronic tongue measure the taste sensitivity of samples and quantifies the sensitivity to express in numerical values [30,32]. Samples were prepared based on the instruction provided by the manufacturer (E-tongue, ASTREE II, Alpha M.O.S, Toulouse, France). The module consists of two reference sensors, SPS and GPS, for calibration as well as the 5 basic tastes: SRS (sourness), STS (saltiness), UMS (umami), SWS (sweetness) and BRS (bitterness). A sample of 10 mL in a 250 mL flask was mixed thoroughly with 90 mL of deionized water for 10 min. The mixture was placed in the module and the analysis was run for 7 replicates. Sensor responses were then transformed to numerical values between 1 and 12 with overall taste descriptions. The responses were presented in a radar plot, which corresponds to relative comparisons of the tastes [28,29,30].

### 2.4. Statistical Analyses

Frying oil samples were analyzed in triplicates and the data were expressed as mean ± standard deviation. Tukey’s multiple range test was applied for comparing means using SAS 9.2 (Statistical Analysis System, Version 9.0, SAS Institute Inc., Cary, NC, USA) at a significance of *p* < 0.05. Pearson’s correlation coefficient was used to estimate correlations between tastes and volatiles at *p* < 0.05 using the Statistical Analysis System software (SAS, ver. 15.1, SAS Institute Inc., Cary, NC, USA). PCA was performed to discriminate the tastes and volatiles identified in the frying oils samples utilizing XLSTAT Software (XLSTAT, version 2019, Addinsoft, New York, NY, USA).

## 3. Results

### 3.1. Chemical Properties

Chemical properties of soybean and canola oils fried up to 20 times are described in Table 1. Oxygen induction time is a core measure of oxidative stability and therefore a critical parameter in establishing shelf life and quality conditions of edible oils [23]. Fresh oils gradually captivate oxygen till oxygen saturation peaks before deterioration and then turns to show a dramatic increase of the capture, which is defined as oxygen induction time [11,23]. Oxygen induction time is largely governed by external factors such as temperature, antioxidants, and saturation degree. It is also inversely related to the degree of unsaturation, indicating that higher the oxidation induction time the higher the oxidative stability [11,23]. As shown in the table, fresh canola oil (4.93 ± 0.10 hr) showed a longer induction time than fresh soybean oil (3.02 ± 0.04 hr). With the increased frying, soybean and canola oils showed a sharp decrease at 5 times (*p* < 0.05) and marked the shortest induction at 20 times, 0.81 ± 0.04 hr and 1.39 ± 0.19 hr, respectively. This result suggested that oxidative stability is compromised due to the repeated frying process and particularly, that canola oil has a higher oxidative stability compared to soybean oil. This may be attributed to the differences in fatty acid compositions between the oils. Canola oil contains a higher amount of oleic acid in comparison to soybean oil. Oleic acid is a monounsaturated fatty acid that is less prone to oxidation during the frying process compared to polyunsaturated fatty acids [33]. Acid value is also an important parameter of estimating oil rancidity. Acid value determines the amount of free fatty acids dissociated from fatty acid chains, and higher acid value indicates a higher degree of deterioration [22,23,24]. Increased acid value not only indicates degradations of the quality of edible oils but negatively influences sensory quality by adding sourness [23]. In the current study, fresh soybean and canola oils showed the lowest acid value, 0.09 ± 0.01 and 0.06 ± 0.02 mg KOH/g, respectively. As the frying time increased, the acid value showed a marked increase at 5 times and 10 times in soy bean and canola oils, respectively (*p* < 0.05), and reached the highest value at 20 times, 0.52 ± 0.01 and 0.44 ± 0.01 mg KOH/g. Canola oil possessed a lower acid value than soybean oil, indicating that canola oil is relatively less prone to deterioration. *p*-anisidine value estimates secondary products induced by lipid oxidation. Increased *p*-anisidine value means an increased amount of secondary oxidation products generated, indicating a higher susceptibility to deterioration [23,24]. As shown in the table, fresh canola oil (17.52 ± 2.30) had higher *p*-anisidine value than fresh soybean oil (16.20 ± 1.63). Repetitive frying processes resulted in a significant increase of *p*-anisidine value (*p* < 0.05) and achieved the maximum at 20 times, 40.26 ± 1.81 and 44.48 ± 1.22 for soybean and canola oils, respectively. Malondialdehyde is a reactive organic compound generated by lipid peroxidation and can be a key indicator in monitoring the extent of lipid oxidation [27]. Higher amounts of malondialdehyde indicates degraded quality of oils. In the present study, fresh soybean oil (0.18 ± 0.02 meq/kg) showed higher malondialdehyde value in comparison to fresh canola oil (0.10 ± 0.02 meq/kg), indicating higher oxidative stability based on higher content of oleic acids in canola oil at ambient temperature compared to soybean oil [33]. As the frying process started however, the increase of malondialdehyde in canola oil was much higher than soybean oil (*p* < 0.05). In general, release of organic compounds during frying is prospectively associated with the frying temperature, and the release substantially increases when reaching its smoke point [34]. Smoke point refers to the temperature where oils start to burn with visible smoke and undergo a series of chemical reactions to produce potentially harmful compounds [35]. Since canola oil possesses a smoke point (180–205 °C) closer to the frying temperature of the present study (180 °C) compared to soybean oil (235–255 °C) [4], the higher amount of malondialdehyde release is expected to be observed in canola oil when the frying initiated. It finally reached its highest at 20 times, 1.65 ± 0.00 and 2.82 ± 0.04 meq/kg for soybean and canola oils, respectively. Total polar compound is another reliable measure to examine deterioration induced by lipid oxidation [11,23]. The result of this study showed a similar pattern with the other indicators. In terms of proportions, fresh soybean (9.67 ± 0.04%) showed about 2-fold higher total polar compounds than fresh canola oil (4.67 ± 0.12%). Again, a high proportion of double bonds present in polyunsaturated fatty acids in soybean oil is more susceptible to chemical transformation during lipid oxidation, leading to the generation of a relatively higher amount of total polar compounds in soybean oil than canola oil [33]. As the repeated frying proceeded, soybean oil did not show statistically significant differences at 5, 10, and 15 times while canola oil showed a significant increase (*p* < 0.05). Total polar compounds exhibited the highest proportion at 20 times, 13.01 ± 0.02% for soybean oil and 8.67 ± 0.24% for canola oil.

All oxidation parameters described in this study increased as the number of frying processes increased. Based on the results of induction time, acid value, and total polar compounds, canola oil is less susceptible to lipid oxidation in comparison to soybean oil when repetitive frying processes are applied. Literatures have also reported oxidation measures with different samples at varied frying conditions; soybean oil and wasabi-added soybean oil showed increased acid value and *p*-anisidine value with potatoes with 10 times frying [36]; acid value, *p*-anisidine value, and total polar compounds significantly increased when chickens were fried 130 rounds at 180 °C [37]; total polar compounds and polymeric triglycerides in palm oil increased when potatoes were fried up to 45 cycles at 180–185 °C [38]; acid value and peroxide value in canola oil, soybean oil, palm oil, and lard increased when pork cutlets and potatoes were fried up to 100 times at 180 °C [10].

### 3.2. Sensory Properties

Volatile compounds of soybean and canola oils fried multiple times were analyzed using an electronic nose to investigate sensory properties and the results are described in Table 2. For soybean oil, 6 compounds were identified in total, among which ethyl butyrate, 2-heptenal, and 2,4-pentadinone were the major compounds. Esters, aldehydes, and ketones are volatile organic compounds commonly released during frying of edible oils and associated with undesirable sensory attributes such as greasiness, off-flavor, and rancid smell [34]. Ethyl butyrate initially presented in low concentration (20.66 ± 1.88) and substantially increased to 130.24 ± 9.42 at 5 times. Then, the increase was not as steep as at 5 times but gradually increased to 260.49 ± 18.90 at 20 times in a statistically significant manner (*p* < 0.05). Ethyl butyrate is an ester eliciting citrus fruity odors associated with apple, apricot, plum, pineapple, and tangerine and is used as a flavoring ingredient [39,40]. Such remarkable increase in ethyl butyrate by the repeated frying may further lead to an increase in sourness, presumably degrading the sensory quality of cooking oils. The concentration of 2-heptenal remarkably increased to 100.25 ± 8.56 at 5 times from the initial (36.44 ± 1.93) (*p* < 0.05) and the increase became mild from 10 times, after which reached highest at 20 times (135.34 ± 13.90). 2-heptenal is an aldehyde easily found in soybean oil and associated with soapy and oily odors [40]. Increased 2-heptenal induced by multiple times of frying may bring an increase of soapy and oily odors, which would give an unfavorable impact on the quality of cooking oils. The concentration of 2,4-pentadienone was initially found 38.23 ± 3.25 and significantly increased to 115.47 ± 10.99 at 15 times (*p* < 0.05). 2,4-pentadienone was then gradually increased with the repeated frying but a statistically insignificant manner. For canola oil, a total of 6 volatile compounds were identified and ethyl butyrate and linalool were found as key compounds. Ethyl butyrate initially existed in low concentrations (18.51 ± 1.12) and significantly increased up to 15 times (*p* < 0.05), after which it reached maximum at 20 times (173.53 ± 17.02). While canola oil exhibited similar increase patterns of ethyl butyrate with soybean oil, actual concentrations after the repeated frying was initiated were much higher in soybean oil compared to canola oil (e.g., 260.49 ± 18.90 vs. 173.53 ± 17.02 at 20 times). Similarly, linalool started at a low level (16.78 ± 1.21) and significantly increased to 102.55 ± 9.21 at 15 times. Linalool induces floral, greenish, and woody odors and is easily found in herbs, citrus fruits, and woods [40]. The result of current study confirmed that despite some differences in the extent of increase, all volatile compounds identified increasingly compromise the sensory quality of the frying oil as the frying process is repeated. Previous studies also showed consistent results with the present study; the concentration of aldehydes, ketons, and alcohols increased in deep fried potatoes using rapeseed oil [41] and canola oil, coconut oil, safflower, and extra virgin olive oil [34]. High molecular weight compounds probably undergo thermal degradation to low molecular weight compounds during the repetitive frying at high temperatures, which may result in an increased release of undesirable volatile compounds [42]. Future studies are required to clearly define the mechanisms embedded in the changes of volatile compounds upon repetitive frying.

**Figure 1:**
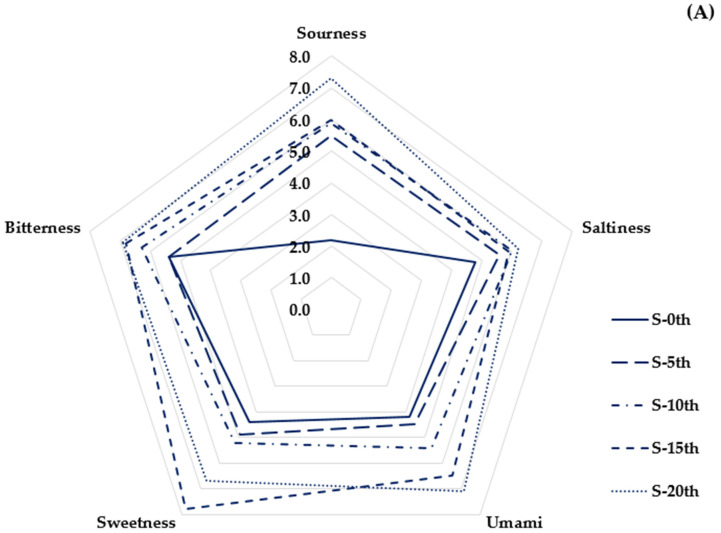

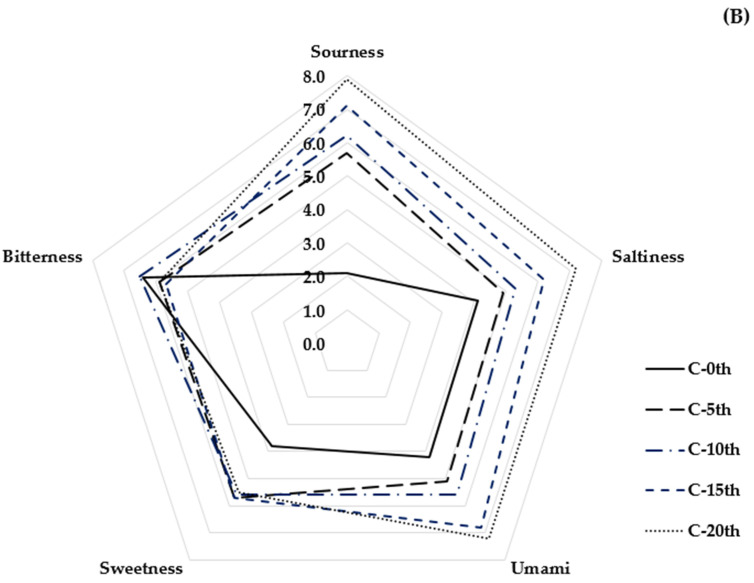
(**A**) Five basic tastes of repetitively fried soybean oil analyzed by electronic tongue. (**B**) Five basic tastes of repetitively fried canola oil analyzed by electronic tongue.

The taste changes of soybean and canola oils that were fried multiple times were analyzed using electronic tongue analysis and the results are depicted in Figure 1A,B. Sourness tended to largely increase in both soybean and canola oils as the frying process repeated. Heightened sourness from acidity is a major deterioration of sensory quality in frying oils. Free fatty acids released from fatty acid chains probably elevate acidity, thereby increasing sourness [11,23]. Bitterness increased in soybean oil but did not show a clear pattern in canola oil. Sweetness gradually increased up to 15 times in soybean oil but without clear patterns as the numbers of frying increased. The umami taste increased in both oils and the increase was slightly higher in canola oil than soybean oil. Similar to umami, saltiness also increased in both oils and the increase was somewhat higher in canola oil compared to soybean oil. The results of this study are consistent with those of a previous study that showed that increased sourness and umami were found in potatoes deep fried 10 times with wasabi-added soybean oil [36]. Further studies are deemed necessary to investigate the amino acid compositions of the oils to understand the various patterns of basic tastes shown in this study.

### 3.3. Multivariate Analyses

Correlations between tastes and volatile compounds were analyzed by Pearson’s correlation coefficients and the results are described in Table 3. Ethyl butyrate positively correlated with sourness (r = 0.82), umami (r = 0.82), and sweetness (r = 0.81) (*p* < 0.05). As aforementioned, ethyl butyrate elicits fruity odors, which may influence this positive correlation between the compound and sourness and sweetness [42]. The positive correlation between ethyl butyrate and sourness coincides with the result of this study that ethyl butyrate and sourness increased with the repeated frying process (Table 2, Figure 1). Linalool positively correlated with sourness (r = 0.8) (*p* < 0.05). Linalool is commonly found in citrus fruits, and is therefore highly associated with sourness, presumably leading to such positive correlation [40]. The sensory results of this study also reflect this correlation that linalool and sourness revealed a significant increase as the numbers of frying increased (Table 2, Figure 1). Interestingly, umami positively correlated with sourness (r = 0.87) and saltiness (r = 0.85) (*p* < 0.05). Development of umami may be attributed to this positive correlation in relation to amino acid compositions at varying conditions. Among volatile compounds, 1-octanol revealed a strong positive correlation with 2,3-pentanedieone (r = 0.99) (*p* < 0.05). This strong positive correlation may derive from oxidation of secondary alcohols to generate ketons when macro-volatiles break down into micro-volatiles during thermal processes [43]. Acetyl pyrazine showed a strong positive correlation with 2,3-pentanedieone (r = 0.97) (*p* < 0.05). This relationship is suggestive of originating from the mechanism of pyrazine formation in the presence of amines and ketons [44].

**Figure 2:**
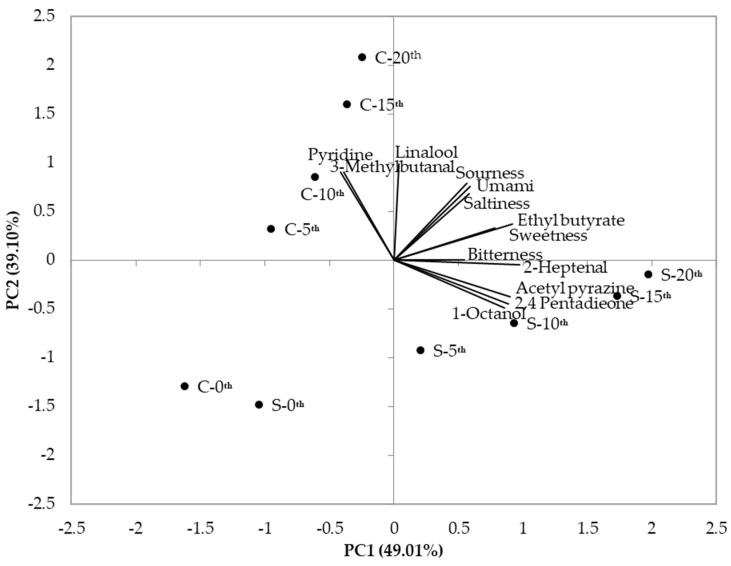
PCA analysis for taste and volatile patterns in repetitively fried soybean and canola oils.

A data set of volatile compounds and basic tastes was subjected to PCA in order to explain the patterns of correlations and the results are plotted in Figure 2. PC1 demonstrates 49.01% of variance in the data set while PC2 demonstrates 39.10%. That is, along PC1, better discrimination was observed in comparison to PC2. In PC1, sourness, saltiness, and umami were at similar rates with each other, indicating a high positive correlation. Ethyl butyrate and sweetness also revealed a high positive correlation. These high positive correlations corresponded to the results of Pearson’s correlation coefficients shown in Table 3. Acetyl pyrazine, 2,4-pentadieone, and 1-octanol were found in similarities with a positive correlation, reflecting the results of correlation analyses (Table 3), but relatively lower discrimination than the correlations mentioned above. These compounds were more closely associated with increased frying in soybean oil than that of canola oil. In PC2, 3-methlybutanal and pyridine exhibited a positive correlation, which is also observed in the correlation analyses (Table 3). These compounds exhibited closer relations to increased frying in canola oil than that of soybean oil. Previous studies used PCA analysis to assess oxidative stability and shelf-life in different types of sunflower oils [45]. Another study demonstrated that six different types of edible oils were successfully segregated with 75% of variance by PCA [46]. Xu et al. (2016) compared cluster analysis (CA), PCA, and linear discriminant analysis (LDA) in order to differentiate oxidized and non-oxidized oils assessed by an electronic nose [47].

## 4. Conclusions

This study examined changes in chemical properties and sensory characteristics of soybean and canola oils with repeated frying. Multivariate analyses were further applied to elucidate correlations among the tastes and key volatile compounds of oils. Repetitive frying at 180 °C resulted in increased levels of chemical parameters including oxygen induction time, acid value, *p*-anisidine value, malondialdehyde, and total polar compounds in both oils. Results of this study suggested that overall, canola oil was chemically more stable during the repetitive frying processes and less prone to lipid rancidity in comparison to soybean oil. Based on the chemosensory and statistical analysis conducted in this study, the chemosensory quality of soybean and canola oils are substantially degraded even with only 5 times of frying. It would be worthwhile for future studies to follow up investigating the quality alterations of these oils with less than 5 times of frying to be able to approximate their shelf-life without compromising sensory quality. The results of this study will serve to give fundamental information to food industry, particularly in controlling the quality of fried foods, evaluating consumer acceptability of fried foods, as well as in product development and cooking methods for fried foods.

## Figures and Tables

**Table 1:** Chemical properties of repetitively fried soybean and canola oils.

Oils	Induction Time (hr)	Acid Value (mg KOH/g)	*p*-Anisidine Value	Malondialdehyde (meq/kg)	Total Polar Compounds (%)
Soybean-0th	3.02 ^a^ ± 0.04 ^1)^	0.09 ^d^ ± 0.01	16.2 ^e^ ± 1.63	0.18 ^e^ ± 0.02	9.67 ^c^ ± 0.04
Soybean-5th	1.86 ^b^ ± 0.06	0.24 ^c^ ± 0.04	23.6 ^d^ ± 0.18	0.54 ^d^ ± 0.02	11.5 ^b^ ± 0.41
Soybean-10th	1.12 ^c^ ± 0.04	0.36 ^b^ ± 0.02	29.0 ^c^ ± 0.34	0.87 ^c^ ± 0.02	11.8 ^b^ ± 0.23
Soybean-15th	0.82 ^d^ ± 0.05	0.47 ^ab^ ± 0.06	34.4 ^b^ ± 1.98	1.24 ^b^ ± 0.04	12.0 ^b^ ± 0.01
Soybean-20th	0.81 ^d^ ± 0.04	0.52 ^a^ ± 0.01	40.3 ^a^ ± 1.81	1.65 ^a^ ± 0.00	13.0 ^a^ ± 0.02
Canola-0th	4.93 ^a^ ± 0.10	0.06 ^e^ ± 0.02	17.5 ^c^ ± 2.30	0.10 ^e^ ± 0.02	4.67 ^d^ ± 0.12
Canola-5th	2.42 ^b^ ± 0.15	0.15 ^d^ ± 0.01	34.1 ^b^ ± 0.87	1.02 ^d^ ± 0.03	6.17 ^c^ ± 0.23
Canola-10th	1.76 ^c^ ± 0.21	0.28 ^c^ ± 0.02	35.1 ^b^ ± 0.69	1.39 ^c^ ± 0.03	7.33 ^b^ ± 0.25
Canola-15th	1.52 ^c^ ± 0.05	0.36 ^b^ ± 0.01	41.0 ^a^ ± 1.14	1.81 ^b^ ± 0.03	8.00 ^ab^ ± 0.10
Canola-20th	1.39 ^c^ ± 0.19	0.44 ^a^ ± 0.01	44.5 ^a^ ± 1.22	2.82 ^a^ ± 0.04	8.67 ^a^ ± 0.24

^1)^ Means with different letters (a–e) within a column in same oil are significantly different (*p* < 0.05).

**Table 2:** Volatile compounds in repetitively fried soybean and canola oils using electronic nose analysis.

Oils	2,4-Pentadinone	Ethyl Butyrate	2-Heptenal	Acetyl Pyrazine	1-Octanol	Linalool	3-Methylbutanal	Pyridine
Soybean-0th	38.23 ^d^ ± 3.25	20.66 ^e^ ± 1.88	36.44 ^c^ ± 1.93	20.32 ^d^ ± 1.56	15.33 ^c^ ± 1.25	17.66 ^d^ ± 1.21	0.00 ± 0.00	0.00 ± 0.00
Soybean-5th	78.22 ^c^ ± 6.28	130.2 ^d^ ± 9.42	100.3 ^b^ ± 8.56	43.41 ^c^ ± 3.32	20.56 ± 2.02	36.13 ^c^ ± 3.01	0.00 ± 0.00	0.00 ± 0.00
Soybean-10th	95.53 ^b^ ± 7.12	200.8 ^c^ ± 14.32	115.9 ^ab^ ± 7.56	50.04 ^b^ ± 4.04	23.23 ^b^ ± 2.06	40.52 ^b^ ± 3.34	0.00 ± 0.00	0.00 ± 0.00
Soybean-15th	115.5 ^a^ ± 10.99	230.4 ^b^ ± 13.97	121.1 ^a^ ± 11.24	53.07 ^b^ ± 3.88	30.51 ^a^ ± 3.11	43.98 ^b^ ± 3.21	0.00 ± 0.00	0.00 ± 0.00
Soybean-20th	118.4 ^a^ ± 8.35	260.5 ^a^ ± 18.90	135.3 ^a^ ± 13.90	60.64 ^a^ ± 3.22	32.38 ^a^ ± 3.69	50.63 ^a^ ± 4.28	0.00 ± 0.00	0.00 ± 0.00
Canola-0th	0.00 ± 0.00	18.51 ^d^ ± 1.12	3.23 ^c^ ± 0.42	5.58 ^e^ ± 0.23	0.00 ± 0.00	16.78 ^d^ ± 1.21	5.03 ^d^ ± 0.34	2.76 ^d^ ± 0.12
Canola-5th	0.00 ± 0.00	85.36 ^c^ ± 6.32	36.34 ^b^ ± 2.54	12.26 ^d^ ± 1.05	0.00 ± 0.00	50.02 ^c^ ± 4.23	25.07 ^c^ ± 1.98	21.67 ^c^ ± 2.86
Canola-10th	0.00 ± 0.00	120.4 ^b^ ± 9.47	47.92 ^ab^ ± 3.18	15.44 ^c^ ± 1.23	0.00 ± 0.00	80.46 ^b^ ± 7.73	28.35 ^b^ ± 3.23	25.82 ^b^ ± 2.13
Canola-15th	0.00 ± 0.00	165.8 ^a^ ± 12.42	55.78 ^a^ ± 4.89	18.08 ^b^ ± 1.47	0.00 ± 0.00	102.6 ^a^ ± 9.21	32.72 ^b^ ± 2.78	29.64 ^a^ ± 2.06
Canola-20th	0.00 ± 0.00	173.5 ^a^ ± 17.02	61.43 ^a^ ± 6.09	22.21 ^a^ ± 1.99	0.00 ± 0.00	110.1 ^a^ ± 8.77	37.87 ^a^ ± 2.22	28.74 ± 2.32

Means with different letters (a–d) within a column in same oil are significantly different (*p* < 0.05).

**Table 3:** Pearson correlation coefficients between taste and flavor compounds in repetitive frying oils.

	Sourness	Saltiness	Umami	Sweetness	Bitterness	2,3-Pentanedione	Ethyl Butyrate	2-Heptenal	Acetyl pyrazine	1-Octanol	Linalool	3-Methylbutanal
Saltiness	0.87 *											
Umami	0.87 *	0.85 *										
Sweetness	0.65	0.52	0.73									
Bitterness	0.24	0.08	0.42	0.57								
2,3-Pentanedione	0.16	0.25	0.18	0.53	0.41							
Ethyl butyrate	0.82 *	0.79	0.82 *	0.81 *	0.53	0.65						
2-Heptenal	0.55	0.59	0.49	0.68	0.40	0.90 *	0.88 *					
Acetyl pyrazine	0.24	0.26	0.20	0.56	0.41	0.97 *	0.69	0.92 *				
1-Octanol	0.09	0.21	0.14	0.50	0.37	0.99 *	0.59	0.87 *	0.95 *			
Linalool	0.80 *	0.73	0.75	0.31	−0.04	−0.40	0.40	0.01	−0.32	−0.44		
3-Methylbutanal	0.49	0.34	0.42	0.01	−0.20	−0.77	−0.05	−0.44	−0.71	−0.80 *	0.87 *	
Pyridine	0.51	0.33	0.43	0.04	−0.20	−0.75	−0.03	−0.42	−0.66	−0.78	0.87 *	0.99 *

Symbol “*” corresponds to significance at *p* < 0.05.

## Data Availability

The data presented in this study are available on reasonable request from the corresponding author.
